# Small Leucine Zipper Protein Regulates Glucose Metabolism of Prostate Cancer Cells via Induction of Phosphoglycerate Kinase 1

**DOI:** 10.3390/cancers16223861

**Published:** 2024-11-18

**Authors:** Sila Han, Sungyeon Park, Suhyun Kim, Sujin Kwon, Jesang Ko

**Affiliations:** Division of Life Sciences, Korea University, Seoul 02841, Republic of Korea; hansila@korea.ac.kr (S.H.); sungyeon10@korea.ac.kr (S.P.); dariakullen@korea.ac.kr (S.K.); kwsu9914@korea.ac.kr (S.K.)

**Keywords:** aerobic glycolysis, metabolic reprogramming, phosphoglycerate kinase 1, prostate cancer, small leucine zipper protein

## Abstract

Background: Cancer cells exhibit altered metabolism whereby glucose is preferentially utilized to produce lactate through aerobic glycolysis. The increase in lactate production creates an acidic microenvironment that supports tumor progression and metastasis. Human small leucine zipper protein (sLZIP) is involved in the transcriptional regulation of genes related to migration and invasion of prostate cancer. However, the role of sLZIP in modulating glucose metabolism in prostate cancer remains unknown. This study investigates whether sLZIP regulates the transcription of glycolysis-related genes to promote metabolic reprogramming in prostate cancer. Methods: Depletion of sLZIP resulted in the downregulation of several glycolytic genes, including glucose transporter 1, phosphofructokinase liver type, phosphoglycerate kinase 1 (PGK1), and lactate dehydrogenase. Among these, only PGK1 showed a prominent dose-dependent decrease in mRNA and protein expression after sLZIP silencing. Results: Mechanistically, increasing or decreasing sLZIP affected the promoter activity of PGK1 in a similar manner. Moreover, the absence of sLZIP attenuated the maximum glycolytic rate in prostate cancer cells. These results were further supported by a reduction in lactate secretion, glucose uptake, and ATP production in sLZIP-knockout prostate cancer cells. sLZIP deficiency hindered cancer growth, as demonstrated by proliferation assays. However, overexpression of PGK1 in sLZIP knockout cells resulted in recovery of aerobic glycolysis. Results of the xenograft experiment revealed that mice injected with sLZIP knockout cells exhibited a decrease in tumor mass compared to those injected with control cells. Conclusion: These findings suggest that sLZIP contributes to the metabolic reprogramming of prostate cancer cells via the transcriptional regulation of PGK1.

## Simple Summary

Prostate cancer is the second leading cause of cancer-related deaths among men, and its recurrence rate is notably high. Depending on the initial treatment, up to 60% of men may experience recurrence within 5 to 10 years after first-line therapy. Our study aims to highlight a novel mechanism for prostate cancer therapy. Human small leucine zipper protein (sLZIP) regulates metabolic reprogramming in prostate cancer, which plays a crucial role in the growth of recurrent prostate cancer. In this study, we found that sLZIP contributes to the metabolic reprogramming of prostate cancer cells via transcriptional regulation of phosphoglycerate kinase 1. The absence of sLZIP attenuated the maximum glycolytic rate and reduced lactate secretion, glucose uptake, and ATP production in prostate cancer. However, overexpression of PGK1 in sLZIP knockout cells resulted in recovery of aerobic glycolysis. sLZIP demonstrates the potential for the development of novel therapeutic approaches and prognostic biomarkers for castration-resistant prostate cancer.

## 1. Introduction

Aerobic glycolysis is a hallmark of cancer and refers to a metabolic shift characterized by increased glycolysis, even in the presence of abundant oxygen, converting glucose into lactate [1]. Unlike normal cells that predominantly utilize oxidative phosphorylation to produce their primary energy source, adenosine triphosphate (ATP), cancer cells display a high preference for glycolysis, which is less efficient for ATP production [1]. Therefore, cancer cells must engage in accelerated glycolysis to compensate for low ATP yield and meet the elevated energy demands of rapidly proliferating cells [2]. In addition to ATP production, the glycolysis pathway provides intermediates essential for the biosynthesis of macromolecules, such as nucleotides, amino acids, and lipids, thereby facilitating tumor growth and survival [3]. Furthermore, an increase in glycolysis results in the conversion of glucose into lactate, which is released into the tumor microenvironment (TME) [4]. This creates an acidic milieu that creates an immunosuppressive TME, impairing the function of immune cells and promoting the migration, invasion, and metastasis of tumor cells [5].

In cancer tissues, many metabolic enzymes of the glycolytic pathway are often dysregulated, and their modification leads to heightened glycolytic flux, which affects cancer metastasis and progression [6]. Key enzymes in glycolysis such as glucose transporter 1 (GLUT1), hexokinase 2 (HK2), phosphoglycerate kinase 1 (PGK1), pyruvate kinase M2 (PKM2), lactate dehydrogenase A (LDHA), and monocarboxylate transporter 4 (MCT4) have been observed to be highly expressed in cancer tissues [7]. GLUT1 upregulation promotes cancer cell proliferation and metastasis by increasing aerobic glycolysis in multiple cancer types, including hepatocellular, breast, and renal cancers [8,9,10]. HK2 is elevated in malignant tumors and promotes the glycolytic phenotype in cancers [11]. Overexpression of MCT4 aids in the efflux of lactate into the extracellular space, contributing to the acidification of the TME and immune evasion [12]. Therefore, altered regulation of glycolytic enzymes in cancer cells contributes to a shift toward aerobic glycolysis.

PGK1 is a key enzyme of the glycolysis pathway that catalyzes the conversion of 1,3-bisphosphoglycerate to 3-phosphoglycerate, generating a molecule of ATP in the process [13]. PGK1 and PKM2 are the only two enzymes that control ATP production during aerobic glycolysis in cancer cells [13]. PGK1 has been reported to be upregulated in various cancer types, such as lung and colon cancers [14,15,16]. PGK1 is essential for the continuation of glycolysis to support the rapid proliferation of cancer cells. Recent studies suggest that increased PGK1 activity plays a role in cancer metastasis and invasiveness [17]. Therefore, PGK1 has emerged as a promising target for therapeutic intervention in cancer, with ongoing research focused on developing strategies to inhibit its function or reduce its expression [18]. However, in-depth research focused on the role and regulation of PGK1 in prostate cancer metabolism has not been extensively explored and remains to be elucidated.

The small leucine zipper protein (sLZIP), an isoform of LZIP (also known as CREB3), functions as a negative regulator of the glucocorticoid receptor (GR), repressing genes in the GR transactivation pathway by recruiting histone deacetylases (HDACs) [19,20]. sLZIP is elevated in cervical cancer and contributes to migration and invasion via the induction of matrix metalloproteinase (MMP)-9 and c-Jun expression [21]. sLZIP is also known to regulate cyclin D3 by directly binding to the AP-1 region of the promoter in prostate cancer to positively regulate tumor growth [22]. However, the role of sLZIP in modulating glucose metabolism through the regulation of glycolytic enzymes in prostate cancer remains unclear. In this study, we investigated the role of sLZIP in the regulation of glucose metabolism in prostate cancer.

## 2. Materials and Methods

### 2.1. Materials

Roswell Park Memorial Institute (RPMI) 1640 medium was purchased from Thermo Fisher Scientific (Waltham, MA, USA). Keratinocyte basal medium 2 and its supplement pack were purchased from PromoCell (Heidelberg, Germany). Fetal bovine serum (FBS) was obtained from HyClone Laboratories (Logan, UT, USA). Myco-Guard™ was obtained from Biomax (Seoul, Republic of Korea). Mouse monoclonal antibodies recognizing β-actin and PGK1 were obtained from Santa Cruz Biotechnology (Santa Cruz, CA, USA). Rabbit polyclonal CREB3 antibody was purchased from Proteintech (Chicago, IL, USA). The anti-mouse and anti-rabbit peroxidase-conjugated secondary antibodies were purchased from Pierce (Madison, WI, USA). Human small interfering RNA (sLZIP) was obtained from GenePharma (Shanghai, China).

### 2.2. Cell Culture and Transfection

PWR-1E, 22Rv1, LNCaP, DU145, and PC3 cells were purchased from ATCC (Manassas, VA, USA). 22Rv1, LNCaP, DU145, and PC3 cells were cultured in RPMI 1640 medium supplemented with 10% FBS and 1% penicillin/streptomycin in a 37 °C humidified incubator containing 5% CO_2_. PWR-1E cells were grown in keratinocyte basal medium 2 with the supplement pack in a 37 °C humidified incubator containing 5% CO_2_. All the cell lines were authenticated using the short tandem repeat (STR) method before use. Cells were transfected with plasmids using the D-fection™ transfection reagent (Lugen Sci, Seoul, Republic of Korea) and incubated for 24 h. Cells were transfected with siRNA using INTERFERin (Polyplus, New York, NY, USA) according to the manufacturer’s protocol.

### 2.3. Stable Cell Line Generation

To establish an sLZIP knockout cell line using the CRISPR/Cas9 system, PC3 cells were transfected with pRGEN-Cas9-CMV or pRGEN-sgRNA (ToolGen Inc., Seoul, Republic of Korea). Stable cell populations were selected using 2 μg/mL puromycin, and the genomic DNA of each clone was analyzed by performing the T7E1 assay. PC3 control (sg-Con) and sLZIP knockout cells (sg-sLZIP #1 and #2) were maintained in RPMI supplemented with 10% FBS, 1% penicillin/streptomycin, and 1 μg/mL puromycin in a 37 °C humidified incubator containing 5% CO_2_. All cell lines were grown in media containing the mycoplasma elimination reagent.

### 2.4. RNA Isolation and Quantitative RT-PCR (qRT-PCR)

Total RNA was isolated using a TaKaRa MiniBEST Universal RNA Extraction Kit (Takara Bio Inc., Kusatsu, Shiga, Japan) according to the manufacturer’s protocol. The cDNA was synthesized from total RNA using the PrimeScript™ RT Master Mix (Takara Bio Inc.). The qRT-PCR was performed on a QuantStudio3 instrument (Thermo Fisher Scientific) using Evagreen Express 2× qPCR Master Mix (Applied Biological Materials, Vancouver, BC, Canada). β-actin was used as an internal control. The primer sequences used for qRT-PCR are listed in Table 1.

### 2.5. Western Blot Analysis

Cells were washed with PBS and lysed in RIPA buffer (150 mM NaCl, 1% Nonidet P-40, 0.1% SDS, 50 mM Tris-HCl, pH 7.4, and protease inhibitors) for 20 min on ice and centrifuged at 12,000× *g* for 20 min at 4 °C. Equal amounts of proteins from each sample were subjected to 10% SDS-PAGE and subsequently transferred onto nitrocellulose membranes. The membranes were probed with specific primary antibodies at 4 °C overnight. β-actin was used as an internal control. The blots were incubated with secondary antibodies for 1 h at 25 °C. The immune complex was detected using the WestGlow™ ECL Chemiluminescent Substrate (Biomax).

### 2.6. Enzyme-Linked Immunosorbent Assay (ELISA)

For the enzyme-linked immunosorbent assay (ELISA), 1 × 10^6^ cells were washed with PBS and lysed in RIPA lysis buffer with protease inhibitors for 30 min on ice and centrifuged at 7000× *g* for 10 min at 4 °C. The activity of PGK1 was measured using the Human PGK1 ELISA Kit (MyBioSource, Inc., San Diego, CA, USA) according to the manufacturer’s instructions. Cells were incubated with TMB substrate for 20 min at 37 °C in dark and absorbance was measured at 450 nm using the microplate reader (Molecular Devices, San Jose, CA, USA).

### 2.7. Plasmid Constructs

For the luciferase assay, the human PGK1 promoter was isolated from genomic DNA obtained from PC3 cells. The promoter region was inserted into a pGL4.21 plasmid vector (Promega, Madison, WI, USA). For transfection, the human PGK1 sequence was inserted into the pCMV-3tag-1A plasmid vector (Agilent Technologies, Santa Clara, CA, USA).

### 2.8. Luciferase Activity Assay

The luciferase activity assay was performed using the Dual-Luciferase Reporter Assay System (Promega). Briefly, PC3 cells were seeded at a density of 1.5 × 10^5^ cells/well. The next day, cells were transfected with the recombinant pGL4.21-PGK1 (−524/−14) promoter plasmid vector and the pRL-CMV *Renilla* plasmid vector to normalize the promoter luciferase activity. The cells were washed with cold phosphate-buffered saline (PBS) and lysed with cell lysis buffer 24 h after transfection. Luciferase activity was measured using a Luminometer 20/20^n^ (Turner BioSystems, Sunnyvale, CA, USA) according to the manufacturer’s protocol.

### 2.9. Extracellular Acidification Rate (ECAR)

PC3 cells were seeded at a density of 5 × 10^4^ cells/well in XF 24-well plates and the medium was replaced with Seahorse assay medium (Agilent Technologies). Cells were treated with the indicated drugs sequentially and ECAR was measured using a Seahorse XF24 Extracellular Flux Analyzer (Agilent Technologies) according to the manufacturer’s protocol. Data were analyzed using the Seahorse Wave.

### 2.10. Glucose Uptake and Lactate Secretion Assays

The supernatant of cultured cells (1.5 × 10^5^ cells/well) was collected after 24 h of incubation and deproteinized using 10 kDa molecular weight cut-off spin columns (Merck KGaA, Darmstadt, Germany). The amount of lactate secreted into the supernatant was measured using a PicoSense™ Lactate Assay Kit (Biomax) according to the manufacturer’s protocol. The absorbance was measured at 570 nm using a microplate reader (Molecular Devices) and normalized to the cell number. Glucose uptake was determined using a Glucose Assay Kit (Merck KGaA) according to the manufacturer’s protocol. The absorbance was measured at 540 nm using a microplate reader (Molecular Devices) and the results were normalized to protein concentrations.

### 2.11. Measurement of ATP

Cells were seeded at a density of 1 × 10^6^ cells/well and cultured in complete media for 24 h. Before measurement of cellular components, cells were homogenized with the assay buffer, and the samples were deproteinized using 10 kDa molecular weight cut-off spin columns (Merck KGaA). Intracellular ATP levels were detected using a colorimetric ATP Assay Kit (Biomax) according to the manufacturer’s protocol. The absorbance was measured at 570 nm using a microplate reader (Molecular Devices).

### 2.12. Cell Viability and Colony Formation Assays

For the cell viability assay, PC3 cells were seeded in triplicate at a density of 5 × 10^3^ cells/well in 96-well plates. A Quanti-MAX™ Cell Viability Assay Kit (Biomax) was used according to the manufacturer’s instructions. After incubating the cells with the Quanti-MAX™ reagent for 30 min, absorbance was measured at 450 nm using a microplate reader (Molecular Devices). For the colony formation assay, 1000 cells were seeded in 12-well plates and incubated for 6 days. Colonies were fixed with 4% paraformaldehyde at 25 °C and stained with 0.05% crystal violet (Millipore Sigma, Burlington, MA, USA). Colony numbers were evaluated by randomly selecting five different areas of the plate.

### 2.13. Animal Study

Six-week-old male BALB/c nude mice were purchased from Orient Bio, Inc. (Seongnam, Republic of Korea). The mice were maintained at 22 ± 2 °C and 50 ± 10% humidity under a 12 h light:12 h dark regimen. The Institutional Animal Care and Use Committee of Korea University approved this study, which was performed according to the Guidelines for the Care and Use of Laboratory Animals. For the xenograft models, the sg-control (1 × 10^6^) and sg-sLZIP (1 × 10^6^) PC3 cells were subcutaneously injected into mice (*n* = 5~6). Twelve days after inoculation, the tumor volume was measured once every two days using a digital caliper and calculated according to the following equation: V = (width^2^ × length) × 0.5. Approximately 33 days post-injection, mouse body weight was measured, and following euthanasia, the tumors were excised, and their weights were recorded.

### 2.14. Histological and Immunohistochemical Analyses

Isolated tumors were incubated with 4% PFA at 4 °C overnight and washed with PBS. The fixed tumors were then dehydrated with ethanol and embedded in the optimal cutting temperature (OCT) compound. The tumor tissues were sectioned at 5-μm thickness using a CM1950 (Leica, Nussloch, Germany) at −20 °C. The OCT compound was then removed using ethanol. For immunohistochemical analysis, tissue slices were prepared using PGK1 or sLZIP antibodies and an ABC kit (Vector Laboratories, Newark, CA, USA). Color was developed using a DAB solution (Vector Laboratories) for up to 1 min. Nuclei were counterstained with Mayer’s hematoxylin for 2 min. Positively stained areas were quantified using ImageJ software 1.54k.

### 2.15. Ethics Statement for Animal Study

We affirm that this study adhered to the Animal Research: Reporting of In Vivo Experiments (ARRIVE) principles. We adhered to the essential procedures specified in the ARRIVE guidelines to minimize suffering and ensure appropriate care and welfare of the experimental animals. The animal studies were approved by the Ethics Committee of Animal Experiments at Korea University (KUIACUC-2023-0086) and all procedures were performed in strict accordance with the Korea University Guide for the Care and Use of Animals in Laboratory Experiments. Animals were euthanized in strict accordance with ethical guidelines. CO_2_ was injected into the chamber at a rate of 30–70% charging per minute. Even after death was visually confirmed, complete euthanasia was induced by exposing the mice to CO_2_ for an additional minute, and death was confirmed by monitoring their heartbeats.

### 2.16. Statistical Analysis

Data are presented as the Mean ± SEM. Statistical evaluation was performed using the GraphPad Prism Software 5 (GraphPad Software, La Jolla, CA, USA). For the two-tailed Student’s *t*-test, values of *p* ≤ 0.05 were considered statistically significant.

## 3. Results

### 3.1. sLZIP Regulates the Expression of PGK1 in Prostate Cancer

**Figure 1:**
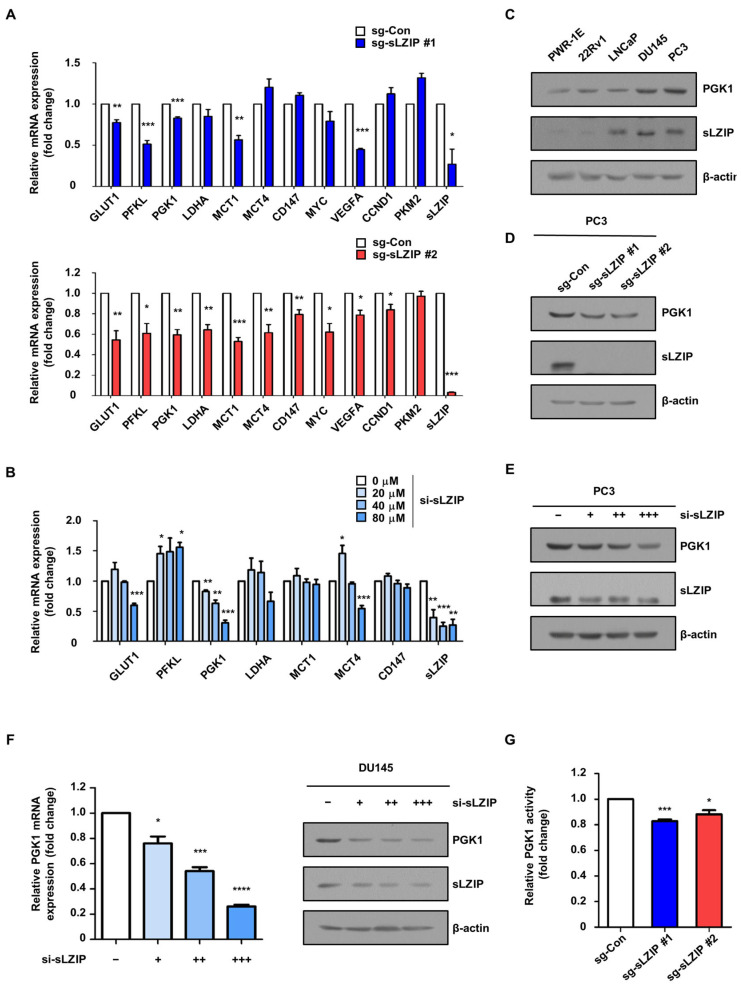
sLZIP regulates the expression of PGK1 in prostate cancer. (**A**) The mRNA expression level of glycolytic genes in sLZIP knockout PC3 cells (sg-sLZIP #1 and #2) was determined using qRT-PCR. β-actin was used as an internal control. (**B**) PC3 cells were transfected with increasing si-sLZIP (0, 20, 40, and 80 μM). mRNA levels were determined using qRT-PCR. β-actin was used as an internal control. (**C**) Protein expression levels were determined by western blotting using whole-cell lysates of PWR-1E, 22Rv1, LNCaP, DU145, and PC3 cells. β-actin was used as an internal control. (**D**) Protein levels of sLZIP knockout PC3 cells were determined by western blotting. β-actin was used as an internal control. (**E**) PC3 cells were transfected with increasing si-sLZIP (0, 20, 40, and 80 μM). Protein levels were determined by western blotting. β-actin was used as an internal control. (**F**) DU145 cells were transfected with increasing si-sLZIP (0, 25, 50, and 100 μM). mRNA and protein levels were examined using qRT-PCR and western blotting, respectively. β-actin was used as an internal control. (**G**) The human PGK1 enzyme activity in sLZIP knockout PC3 cells was measured through ELISA. All *p*-values were obtained using unpaired two-tailed Student’s *t*-test. * *p* < 0.05, ** *p* < 0.01, *** *p* < 0.001, **** *p* < 0.0001. Error bars: Mean ± SEM of three experiments.

Prostate cancer cells exhibit a reprogrammed metabolism, whereby aerobic glycolysis is preferred even in the presence of abundant oxygen, allowing cancer cells to generate ATP to support uncontrolled growth and proliferation [23]. We investigated whether sLZIP is involved in the metabolic reprogramming of prostate cancer cells. To investigate the role of sLZIP in the regulation of glycolytic gene expression, we generated a sLZIP-knockout PC3 cell line using the CRISPR/Cas9 system. The sLZIP-knockout PC3 cell lines (sg-sLZIP #1 and #2) showed decreased mRNA expression of several glycolytic genes, including GLUT1, PFKL, PGK1, and LDHA, compared to PC3 control cells (Figure 1A). In addition, sLZIP knockout cells showed a decrease in the mRNA expression levels of genes related to lactate transport, such as MCT1 and MCT4, and the cell surface glycoprotein CD147, compared to the control cells (Figure 1A). To determine the role of sLZIP in the regulation of these genes, we transiently silenced sLZIP using si-sLZIP. Of the aforementioned genes, only PGK1 showed a dose-dependent decrease in mRNA expression in the PC3 cells (Figure 1B). Since both knockout and transient silencing of sLZIP in the PC3 cells resulted in a decrease in PGK1 mRNA expression, we further analyzed the effect of sLZIP deprivation on PGK1 expression in prostate cancer. First, we examined the protein expression levels of sLZIP and PGK1 in several prostate cell lines, including PWR-1E, 22Rv1, LNCaP, DU145, and PC3. The results revealed a progressive increase in both sLZIP and PGK1 protein levels as prostate cancer progressed. The expression level of PGK1 was elevated in androgen-independent DU145 and PC3 cells (Figure 1C). The sLZIP knockout decreased PGK1 protein expression compared to that in the control PC3 cells (Figure 1D). The si-sLZIP decreased PGK1 expression in a dose-dependent manner in the PC3 cells (Figure 1E). Furthermore, transient silencing of sLZIP resulted in a dose-dependent decrease in PGK1 mRNA and protein expression in the DU145 cells (Figure 1F). We also determined the enzymatic activity of PGK1. The sLZIP-knockout PC3 cells showed a decrease in PGK1 enzyme activity compared to the control PC3 cells (Figure 1G). These results indicated that sLZIP regulates the expression level of PGK1 in prostate cancer.

### 3.2. sLZIP Functions as a Transcriptional Regulator of PGK1 in Prostate Cancer

**Figure 2:**
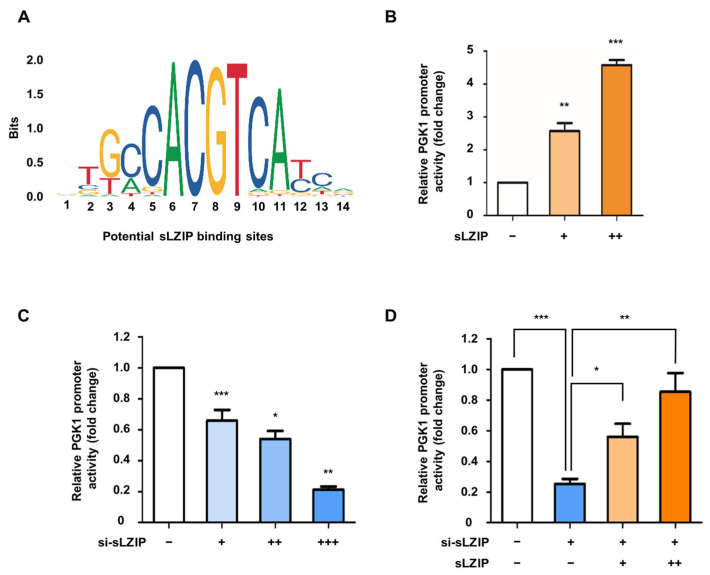
sLZIP functions as a transcriptional regulator of PGK1 in prostate cancer. (**A**) JASPAR enrichment analysis of potential sLZIP binding sites. (**B**) PC3 cells were transfected with the human PGK1 promoter, the Renilla-encoded vector, and increasing GFP-sLZIP expression plasmids (0, 0.25, and 0.5 μg). Promoter activity was measured after 24 h of transfection. (**C**) PC3 cells were transfected with the human PGK1 promoter, the Renilla-encoded vector, and increasing concentration of si-sLZIP (0, 20, 40, and 80 μM). Promoter activity was measured after 24 h of transfection. (**D**) PC3 cells were transfected with the human PGK1 promoter, the Renilla-encoded vector, si-sLZIP (80 μM), and increasing GFP-sLZIP expression plasmids (0, 0.25, and 0.5 μg). Promoter activity was measured after 24 h of transfection. All *p*-values were obtained using unpaired two-tailed Student’s *t*-test. * *p* < 0.05, ** *p* < 0.01, *** *p* < 0.001. Error bars: Mean ± SEM of three experiments.

Since sLZIP acts as a transcription factor in various cellular processes, we examined whether sLZIP regulates the transcription of PGK1 in prostate cancer. First, JASPAR enrichment analysis was used to identify potential sLZIP-binding sites. Three potential binding sites were identified in the human PGK1 promoter (Figure 2A). Therefore, we cloned the PGK1 promoter (−524/−14) region and performed a luciferase assay. The results showed that sLZIP increased PGK1 promoter activity in a dose-dependent manner in the PC3 cells by approximately 4.5-fold (Figure 2B). However, cells transfected with si-sLZIP showed a dose-dependent decrease in PGK1 promoter activity, indicating the need for endogenous sLZIP for the transcription of PGK1 (Figure 2C). Overexpression of sLZIP in sLZIP-knockout cells resulted in the recovery of PGK1 promoter activity (Figure 2D). These findings indicate that sLZIP induces the transcription of PGK1 in prostate cancer.

### 3.3. sLZIP Induces Metabolic Reprogramming of Prostate Cancer Cells and Promotes Cell Proliferation

**Figure 3:**
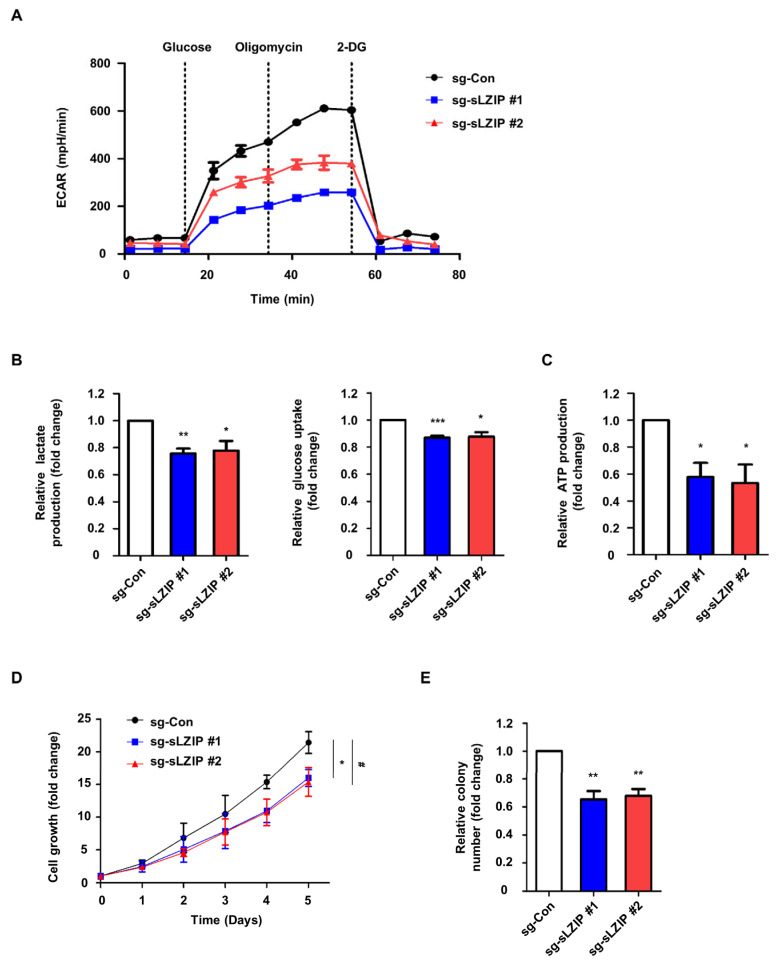
sLZIP induces metabolic reprogramming of prostate cancer cells and promotes cell proliferation. (**A**) The sLZIP knockout PC3 cells were seeded in XF 24-well plates and ECAR levels were measured using XF Analyzer. Raw ECAR data were normalized against the total amount of proteins. ECAR data were quantified using the Seahorse Wave software 2.6. (**B**) Lactate production and glucose uptake levels of sLZIP knockout PC3 cells were measured. Conditioned medium was collected from PC3 control and PC3 sLZIP knockout cells after 24 h in fresh medium. The amount of lactate present in the conditioned medium was analyzed using the lactate assay. The amount of glucose uptake was measured by subtracting the amount of glucose in the original medium and was analyzed through the glucose assay. Results were normalized with cell number and total protein level, respectively. (**C**) ATP production levels were measured in sLZIP knockout PC3 cells. Results were normalized with the total protein level. (**D**) Cell viability of sLZIP knockout PC3 cells was evaluated by MTT assay over 6 days. (* *p*-value; sg-sLZIP #1, # *p*-value; sg-sLZIP #2). (**E**) The colony number of sLZIP knockout PC3 cells was measured by colony formation assay over 5 days. All *p*-values were obtained using unpaired two-tailed Student’s *t*-test. * *p* < 0.05, ** *p* < 0.01, *** *p* < 0.001. Error bars: Mean ± SEM of three experiments.

As sLZIP induces the transcription of PGK1, we investigated whether sLZIP contributes to aerobic glycolysis in prostate cancer. We monitored ECAR as an indicator of glycolysis. The sLZIP knockout cells showed decreased ECAR levels compared to the control cells (Figure 3A). The effects of sLZIP on lactate production and glucose uptake in prostate cancer cells were also examined. The results of the lactate and glucose assays demonstrated that sLZIP knockout reduced lactate production and glucose uptake compared to the control (Figure 3B). ATP assay results showed that sLZIP knockout decreased ATP production compared to the control (Figure 3C). We also investigated the role of sLZIP in cell proliferation and growth. The sLZIP knockout cells showed decreased cell viability compared to the control cells (Figure 3D). In addition, sLZIP knockout compromised the growth of PC3 cells, as demonstrated by colony formation assay (Figure 3E). These results suggest that sLZIP induces metabolic reprogramming of prostate cancer cells and promotes cell proliferation.

### 3.4. Overexpression of PGK1 in sLZIP Knockout Cells Recovered Aerobic Glycolysis

**Figure 4:**
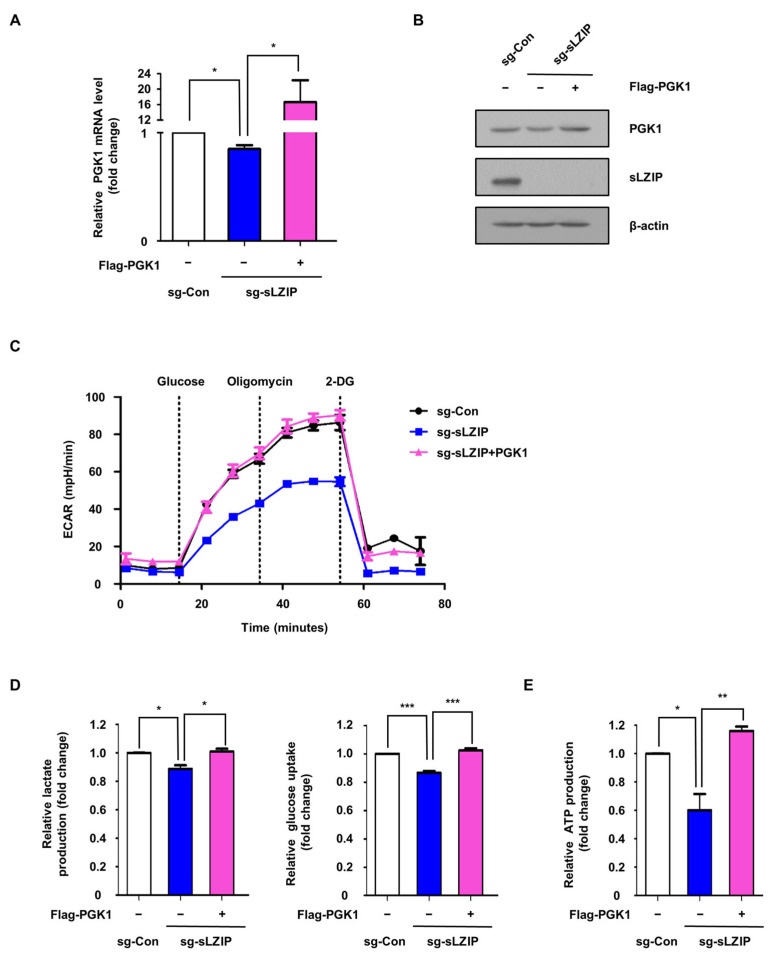
Overexpression of PGK1 in sLZIP knockout cells recovered aerobic glycolysis. (**A**,**B**) The sLZIP knockout PC3 cell (sg-sLZIP #1) was transfected with Flag-PGK1 (1.5 μg) and the following functional assays were performed. The mRNA and protein expression levels were determined through qRT-PCR and western blotting, respectively. β-actin was used as an internal control. (**C**) PC3 cells were seeded in XF 24-well plates and ECAR was measured using the XF Analyzer. Raw ECAR data were normalized against the total amount of proteins. ECAR data were quantified using the Seahorse Wave software 2.6. (**D**) Lactate production and glucose uptake levels were measured. Conditioned medium was collected after 24 h in fresh medium. The amount of lactate present in the conditioned medium was analyzed using the lactate assay. The amount of glucose uptake was measured by subtracting the amount of glucose in the original medium and was analyzed using the glucose assay. Results were normalized with cell number and total protein level, respectively. (**E**) ATP production levels were measured. Results were normalized with the total protein level. All *p*-values were obtained using unpaired two-tailed Student’s *t*-test. * *p* < 0.05, ** *p* < 0.01, *** *p* < 0.001. Error bars: Mean ± SEM of three experiments.

To verify whether sLZIP promotes aerobic glycolysis by inducing PGK1 expression, we overexpressed PGK1 in sLZIP-knockout PC3 cells. The efficiency of PGK1 overexpression was confirmed by qRT-PCR analysis and western blotting before performing the following assays (Figure 4A,B). ECAR measurements revealed that PGK1 overexpression rescued aerobic glycolysis in sLZIP knockout cells (Figure 4C). Lactate production and glucose consumption assays were performed to validate these results (Figure 4D). In addition, overexpression of PGK1 rescued the decreased ATP production caused by the sLZIP knockout (Figure 4E). Thus, sLZIP promotes aerobic glycolysis in prostate cancer cells by regulating PGK1 expression.

### 3.5. Deletion of sLZIP Inhibits Tumor Growth by Regulating PGK1 Expression in Vivo

**Figure 5:**
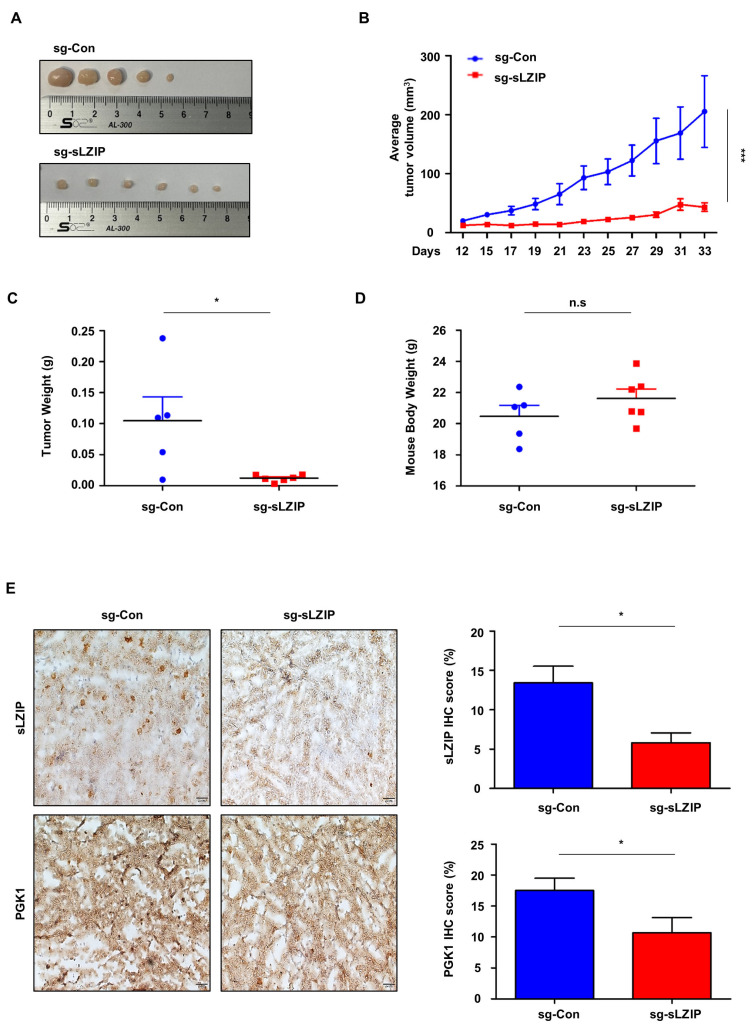
Deletion of sLZIP inhibits tumor growth by regulating PGK1 expression in vivo. Xenograft models were generated by subcutaneously injecting sg-control (1 × 10^6^) and sg-sLZIP (1 × 10^6^) PC3 cells into nude mice. (**A**) Images of the xenografts from the sg-control group (*n* = 5) and sg-sLZIP group (*n* = 6) showing 33 days following cell injection. (**B**) Quantification of tumor volume in mice of the sg-control group and the sg-sLZIP group over time. (**C**,**D**) Tumor weight and body weight are measured for 33 days after cell injection. (**E**) Representative images of immunohistochemical staining for sLZIP and PGK1 in sectioned tumor tissue of the two mice groups (Scale bars, 50 μm). The IHC score was determined for positive areas of immunohistochemical staining. All *p*-values were obtained using unpaired two-tailed Student’s *t*-test. n.s, not significant, * *p* < 0.05, *** *p* < 0.001. Error bars: Mean ± SEM of three experiments.

Since sLZIP promotes aerobic glycolysis and cell proliferation by regulating PGK1 expression in prostate cancer, we performed xenograft experiments using nude mice. Sg-control or sLZIP knockout PC3 cells were subcutaneously injected into nude mice. Tumor size, tumor growth, and tumor weight were reduced in the group injected with sLZIP knockout cells compared to those in the group injected with sg-control cells (Figure 5A–C). However, body weight was not affected due to sLZIP (Figure 5D). Immunohistochemistry results showed that the levels of sLZIP and PGK1 decreased in the group injected with sLZIP knockout cells compared to those in the group injected with sg-control cells (Figure 5E). These results indicate that sLZIP promotes prostate cancer progression by regulating PGK1 expression.

## 4. Discussion

Metabolic abnormalities are a hallmark of cancer, and new treatment therapies targeting enzymes responsible for the increased glucose metabolism have shown great potential in blocking cancer progression [24,25]. Because aerobic glycolysis is the primary source of energy in cancer cells, 70% of human cancers display a ubiquitous overexpression of glycolytic genes [26]. We found that the sLZIP knockout decreased aerobic glycolysis in prostate cancer cells by reducing the transcription of several glycolysis-related genes. Therefore, sLZIP modulates metabolic reprogramming and may serve as a novel therapeutic target for the treatment of prostate cancer.

PGK1 catalyzes the first ATP production step during aerobic glycolysis, allowing cancer cells to maintain glycolytic flux and support the anabolic production of biomolecules for uncontrolled proliferation, especially under hypoxic conditions [27]. We found that sLZIP plays an important role in cancer proliferation and growth through transcriptional regulation of PGK1. sLZIP binds to cAMP response elements (CREs) to activate MMP-9 transcription in cervical cancer and microtubule-associated protein 1A/1B-light chain 3 transcription in colorectal cancer [21,28]. As the PGK1 promoter contains three CRE sites, sLZIP likely binds to the CRE site of the PGK1 promoter. Luciferase assay results revealed that sLZIP exerted regulatory effects on PGK1 promoter activity. PGK1 not only functions as a metabolic enzyme but also as a protein kinase that regulates signal transduction pathways closely related to cell growth, division, and tumor development [29]. In the mitochondria, PGK1 mediates the activation of pyruvate dehydrogenase kinase 1 by direct phosphorylation at Thr338, which in turn phosphorylates pyruvate dehydrogenase, thus inhibiting the pyruvate dehydrogenase complex activity [29]. This hinders the conversion of pyruvate and CoA to acetyl-CoA in the mitochondria, increasing the flow of pyruvate towards conversion of lactate by LDHA, thus promoting aerobic glycolysis and tumor development [29]. Since sLZIP regulates PGK1 expression, it may contribute to the inhibitory effect on mitochondrial respiration and heightened aerobic glycolysis through PGK1 kinase activity.

Cancer cell-derived lactate accumulates in the TME to create an acidic milieu that facilitates tumor invasion and immune escape, which in turn promotes tumor growth [30]. Recent studies have confirmed that tumors with high lactate levels are positively associated with tumor recurrence and metastasis, leading to poor prognosis in patients with cancer [31]. Therefore, targeting lactate production is an important area of research in TME metabolism and cancer development. Our study indicates that sLZIP promotes lactate secretion via PGK1 by enhancing the glycolytic activity of prostate cancer cells. Recent research has revealed that cancer-derived lactate in the TME triggers the polarization of resting M0 macrophages into tumor-associated macrophages (TAMs) and suppresses inflammation and immune cell infiltration [30]. TAMs create an anti-inflammatory TME that promotes the proliferation, migration, and invasion of colorectal tumor cells [32]. Whether lactate induced by sLZIP activates TAMs is yet to be investigated; however, we suggest that the activation of TAMs by sLZIP-induced lactate may predict the possibility of promoting tumor progression, and this is valuable as new research. Moreover, it has been discovered that TAMs polarized by lactate secrete exosomes containing various factors, which, when internalized by cancer cells, enhance epithelial-mesenchymal transition (EMT) [33]. The factors conveyed by TAM-secreted exosomes include signaling molecules, microRNAs associated with tumor progression, growth factors that promote angiogenesis, and cytokines that influence the immune response within the TME [34]. Recent evidence suggests that TAMs enhance the invasion and metastasis of colon cancer cells by secreting interleukin-6 (IL-6) to induce EMT [34,35]. In addition, IL-6 secreted by polarized TAMs enhances the phosphorylation of PGK1 in tumor cells and activates PGK1, demonstrating a novel mechanism by which macrophages promote tumor growth by regulating glucose metabolism [36]. As sLZIP promotes lactate production, it may be involved in TAM polarization, which in turn activates PGK1 in a positive feedback loop.

Previous studies have shown that there is a positive correlation between the level of sLZIP expression and prostate cancer progression [20,22]. In addition, both sLZIP and PGK1 expression are higher in androgen-independent prostate cancer cells compared to androgen-dependent prostate cancer cells. Increased sLZIP may induce PGK1 expression, which may promote aerobic glycolysis and promote prostate cancer progression in the absence of androgen signaling. Therefore, it may be hypothesized that sLZIP-induced PGK1 expression may affect the transition from androgen-dependent prostate cancer to androgen-independent prostate cancer. However, further studies are needed to verify the effect of sLZIP-induced PGK1 expression in androgen-dependent prostate cancer cells.

## 5. Conclusions

sLZIP levels are elevated in androgen-independent prostate cancer cells compared to androgen-dependent prostate cancer cells. sLZIP promotes the transcription of PGK1, leading to lactate accumulation and consequently facilitating the progression of androgen-independent prostate cancer cells. These results indicate that sLZIP contributes to the metabolic reprogramming of prostate cancer cells via the transcriptional regulation of PGK1. Our findings suggest that sLZIP is associated with resistance to androgen-independent conditions. Therefore, sLZIP has emerged as a potential candidate for the development of therapeutic strategies and prognostic markers for androgen-independent prostate cancer.

## Figures and Tables

**Table 1:** The primer sequences used for qRT-PCR.

Target mRNA	Primer Sequence
*GLUT1*	F: 5′-GGCTTCTCCAACTGGACCTC-3′
	R: 5′-CCGGAAGCGATCTCATCGAA-3′
*PFKL*	F: 5′-AAGAAGTAGGCTGGCACGAC-3′
	R: 5′-GCGGATGTTCTCCACAATGG-3′
*PGK1*	F: 5′-ATGCTGAGGCTGTCACTCG-3′
	R: 5′-CACAGCAAGTGGCAGTGTCT-3′
*LDHA-*	F: 5′-CATGGCCTGTGCCATCAGTA-3′
	R: 5′-AGATATCCACTTTGCCAGAGACA-3′
*MCT1*	F: 5′-GTGGCTCAGCTCCGTATTGT-3′
	R: 5′-GAGCCGACCTAAAAGTGGTG-3′
*MCT4*	F: 5′-CGTTCTGGGATGGGACTGAC-3′
	R: 5′-ATGTGCCTCTGGACCATGTG-3′
*CD147*	F: 5′-TGCTGGTCTGCAAGTCAGAG-3′
	R: 5′-GCGAGGAACTCACGAAGAAC-3′
*MYC*	F: 5′-ACACCCTTCTCCCTTCG-3′
	R: 5′-CCGCTCCACATACAGTCC-3′
*VEGFA*	F: 5′-GGGCAGAATCATCACGAAGT-3′
	R: 5′-ATCTGCATGGTGATGTTGGA-3′
*CCND1*	F: 5′-GCGAGGAACAGAAGTGC-3′
	R: 5′-GAGTTGTCGGTGTAGATGC-3′
*PKM2*	F: 5′-CTGCAGTGGGGCCATAATC-3′
	R: 5′-GCCAACATTCATGGCAAAGT-3′
*sLZIP*	F: 5′-AGCAGCAGCATGTACTCCTCT-3′
	R: 5′-AGGCAGCTCCAGCTGGTAAG-3′
*β-actin*	F: 5′-AGCGAGCATCCCCCAAAGTT-3′
	R: 5′-GGGCACGAAGGCTCATCATT-3′

## Data Availability

The data underlying this article will be shared at reasonable request to the corresponding author.
